# Incidence of melanoma in situ among racial and ethnic minorities in the United States

**DOI:** 10.1016/j.jdin.2024.08.015

**Published:** 2024-09-27

**Authors:** Gurman S. Dhaliwal, Adina Greene, Andy Ho, Aaron R. Mangold, Collin M. Costello

**Affiliations:** a Department of Dermatology, Mayo Clinic, Scottsdale, Arizona; b University of Arizona College of Medicine – Phoenix, Phoenix, Arizona; c Mayo Clinic Alix School of Medicine, Scottsdale, Arizona

**Keywords:** epidemiology, incidence rates, melanoma, melanoma in situ, prevalence

*To the Editor:* Recent population-based studies within the United States suggest that overdiagnosis may be contributing to rising melanoma incidences, particularly for melanoma in situ (MIS).^1^, ^2^, ^3^ Melanoma screening initiatives are associated with increased biopsy rates and detection of MIS lesions.^4^^,^^5^ Literature evaluating MIS incidence trends based on patient race/ethnicity is missing. We sought to evaluate MIS incidence rates stratified by race/ethnicity among the US population.

This study utilized the Surveillance, Epidemiology, and End Results database. Based on our inclusion criteria, we analyzed 541,596 melanoma cases between 2004 and 2019 [238,709 MIS and 302,887 cutaneous malignant melanoma (CMM)]. Racial subgroups were subclassified as non-Hispanic White (NHW), non-Hispanic Black (NHB), non-Hispanic American Indian/Alaska Native (NH AI/AN), non-Hispanic Asian or Pacific Islander (NH A/PI), and Hispanic. Surveillance, Epidemiology, and End ResultsStat software (version 8.4.2) was utilized to calculate crude incidence rates and trends based on average annual percent change (AAPC). Supplementary Methods, available via Mendeley at https://data.mendeley.com/datasets/7rsj8pjm72/1 for greater detail.

Between 2004 and 2019, the crude incidence rates for MIS lesions significantly increased by an AAPC of +5.66% among NHWs (*P* < .001), +3.19% among NHBs (*P* = .03), +9.79% among NH AI/ANs (*P* < .001), +2.97 among NH A/PIs (*P* = .01), and +5.19% among Hispanics (*P* < .001) (Table I). Between 2004 and 2010, the crude incidence rates for MIS lesions significantly increased by an AAPC of +6.16% among NHWs (*P* < .001) and +3.42% among Hispanics (*P* = .04); no significant trends were observed among NHBs, NH AI/ANs, nor NH A/PIs. Between 2011-2019, the crude incidence rates for MIS lesions significantly increased by an AAPC of +4.63% among NHWs (*P* < .001), +6.35% among NH AI/ANs (*P* = .02), +5.76% among NH A/PIs (*P* = .01), and +5.94% among Hispanics (*P* < .001); no significant trends were observed among NHBs. See Table I for details on MIS and CMM incidence and AAPC. See Supplementary Table I, available via Mendeley at https://data.mendeley.com/datasets/7rsj8pjm72/1 for annual crude incidence rates.

**Table I:** Crude incidence rates and the corresponding average annual percent change based on racial designation for melanoma cases diagnosed in the United States between 2004 and 2019

Race	2004-2019				2004-2010				2011-2019			
	Incidence rate	AAPC	AAPC 95% CI	AAPC *P*-value	Incidence rate	AAPC	AAPC 95% CI	AAPC *P*-value	Incidence rate	AAPC	AAPC 95% CI	AAPC *P*-value
Melanoma in situ												
NHW	41.58	5.66∗	5.02-6.29	<.001	31.17	6.16∗	5.07-7.27	<.001	49.52	4.63∗	2.86-6.43	<.001
NHB	0.47	3.19∗	0.44-6.02	.03	0.38	1.92	−8.24-13.2	.66	0.52	0.69	−6.62-8.58	.84
NH AI/AN	6.10	9.79∗	7.12-12.53	<.001	3.24	3.61	−7.52-16.07	.46	8.11	6.35∗	1.59-11.33	.02
NH A/PI	0.90	2.97∗	0.91-5.08	.01	0.77	−2.91	−12.81-8.12	.51	0.98	5.76∗	2.25-9.40	.01
Hispanic	2.89	5.19∗	4.40-5.99	<.001	2.21	3.42∗	0.22-6.72	.04	3.32	5.94∗	3.96-7.95	<.001
Cutaneous malignant melanoma												
NHW	52.02	2.67∗	2.39-2.95	<.001	45.84	2.65∗	1.77-3.54	<.001	56.73	2.58∗	1.70-3.47	<.001
NHB	1.22	−0.30	−1.94-1.37	.70	1.24	1.86	−5.20-9.44	.54	1.21	−1.14	−5.55-3.49	.57
NH AI/AN	9.37	4.31∗	2.56-6.09	<.001	7.20	−1.36	−9.79-7.86	.71	10.91	3.26∗	0.03-6.59	.05
NH A/PI	1.83	0.02	−1.27-1.33	.97	1.78	−2.56	−6.37-1.41	.16	1.87	−1.31	−4.86-2.37	.42
Hispanic	4.98	1.71∗	0.81-2.62	<.001	4.52	−1.63	−4.59-1.41	.22	5.28	2.03	−0.13-4.24	.06

Crude incidence rate values are per 100,000 individuals.

*AAPC*, Average annual percent change; *CI*, confidence interval; *NH AI/AN*, Non-Hispanic American Indian/Alaskan Native; *NH A/PI*, Non-Hispanic Asian/Pacific Islander; *NHB*, Non-Hispanic Black; *NHW*, Non-Hispanic White.

∗ The average annual percent change is statistically significant (*P* < .05).

The incidence of MIS increased among each racial/ethnic subgroup from 2004 to 2019. From 2004 to 2010, only Hispanics and NHWs experienced a rise in MIS incidence; however, from 2011 to 2019, there was a rise in MIS incidence in NH AI/ANs, NH A/PIs, and Hispanics at a rate at least equivalent to NHWs. This raises concerns about the growing number of MIS diagnosed in our non-White populations after a period of stable MIS rates.

This study's limitations include retrospective data and self-identified race/ethnicity; its strengths include utilizing a national registry and a large sample size.

Although the overall incidence of MIS is low among non-White populations compared to NHWs, the sharp rise in MIS incidence rates should be monitored closely. If the rising MIS rates among non-White melanoma populations primarily stemmed from catching biologically aggressive melanoma early, one would expect a subsequent decline in CMM incidence rates. However, CMM rates remain unchanged among NHBs and NH A/PIs and are increasing among NH AI/ANs and Hispanics. This suggests that, similar to NHWs, a proportion of MIS diagnosed in our non-White populations may be a direct result of increased surveillance. Minority populations with lower melanoma mortality rates may have a higher risk for overdiagnosis, increased benign-to-malignant biopsy ratios, and associated physical, psychological, social, and financial harm. Further research is needed to understand the rising trend of MIS in non-White populations.

## Conflicts of interest

Dr Costello is an investigator for DeepX Health and Merck. Dr Mangold has consulted for Kyowa, Eli Lilly, Momenta, UCB, and Regeneron in the past, greater than 24 months ago. He has consulted for PHELEC in the past, great than 12 months. He has consulted for Incyte, Soligenix, Clarivate, and Bristol Myers Squibb in the past, less than 12 months ago. He consults for Argenyx, Boehringer, Janssen, and Ingelheim currently. He consults for Regeneron and Pfizer currently with payments to the institution. He received grant support from Kyowa, Miragen, Regeneron, Corbus, Pfizer, Incyte, Eli Lilly, Aregenx, Palbella, Abbvie, Priovant, Merck in the last 24 months. Beyond 24 months, grant support has come from Sun Pharma, Elorac, Novartis, and Janssen. His current patents include Methods and Materials for assessing and treating cutaneous squamous cell carcinoma (provisional 63-423254), use of oral JAKi in Lichen Planus (provisional 63/453065), and Topical Ruxolitinib in Lichen Planus (WO2022072814A1). Dr Dhaliwal and Authors Greene and Ho have no conflicts of interest to declare.

## References

[1] Adamson A.S., Suarez E.A., Welch H.G. (2022). Estimating overdiagnosis of melanoma using trends among Black and White patients in the US. JAMA Dermatol.

[2] Kurtansky N.R., Dusza S.W., Halpern A.C. (2022). An epidemiologic analysis of melanoma overdiagnosis in the United States, 1975-2017. J Invest Dermatol.

[3] Welch H.G., Mazer B.L., Adamson A.S. (2021). The rapid rise in cutaneous melanoma diagnoses. N Engl J Med.

[4] Matsumoto M., Wack S., Weinstock M.A. (2022). Five-year outcomes of a melanoma screening initiative in a large Health care system. JAMA dermatology.

[5] Whiteman D.C., Olsen C.M., MacGregor S. (2022). The effect of screening on melanoma incidence and biopsy rates. Br J Dermatol.

